# Solid pseudopapillary neoplasm of the pancreas with elevated β-HCG levels: A case report

**DOI:** 10.1097/MD.0000000000043808

**Published:** 2025-08-22

**Authors:** Minfen Zhang, Zhi Duan, Zhen Li, Jiao He, Lingli Liang, Meiyan Wei, Ting Tao, Wanli Lin, Hui Chen

**Affiliations:** a Department of Pathology, The First Hospital of Changsha, Changsha, Hunan Province, China.

**Keywords:** pregnancy, solid pseudopapillary pancreatic neoplasms, vaginal bleeding, β-HCG

## Abstract

**Rationale:**

Solid pseudopapillary neoplasm of the pancreas, first classified as a malignant epithelial tumor of the pancreas in 2019, is rare and usually occurs in young women, with a high incidence at approximately 30 years of age. Herein, we present a case of solid pseudopapillary neoplasm of the pancreas with elevated β-human chorionic gonadotropin (HCG) levels, which, to our knowledge, has not been reported in the literature.

**Patient concerns:**

A 35-year-old woman of childbearing age presented with a chief complaint of vaginal bleeding and was admitted to our hospital. Her β-HCG level was elevated; however, a complete endometrial biopsy and related imaging examinations revealed no evidence of pregnancy or trophoblastic neoplasm. Computed tomography revealed a lump of abnormal signals in the head of the pancreas, which was suspected to be a solid pseudopapilloma.

**Diagnoses:**

Hence, surgery was performed, and the postoperative pathological diagnosis of a solid pseudopapillary neoplasm of the pancreas was confirmed.

**Interventions and outcomes:**

The tumor was completely removed by surgery. Notably, the patient’s β-HCG level gradually decreased after removal of the pancreatic mass and returned to normal levels after 1 month.

**Lessons:**

We present a case of solid pseudopapillary neoplasm of the pancreas that was diagnosed using hematoxylin and eosin staining and immunohistochemistry. In our case, the solid pseudopapillary neoplasm partially expressed β-HCG. Further studies are needed to confirm whether β-HCG promotes the occurrence and development of solid pancreatic pseudopapillary neoplasms.

## 1. Introduction

Solid pseudopapillary neoplasms (SPNs) of the pancreas are typically incidentally found and have no specific clinical symptoms. Although their biological behavior is a low-grade malignant tumor,^[1]^ some cases demonstrate malignant behavior with recurrence and metastases.^[2]^ A retrospective study found that approximately half of the patients with SPNs of the pancreas were asymptomatic, whereas others presented with diarrhea and abdominal pain, distension, or masses. Approximately 13% of patients with SPNs of the pancreas have elevated serum tumor markers, including alpha-fetoprotein, carcinoembryonic antigen (CEA), CA125, CA199, CA724, and neuron-specific enolase.^[3]^ β-Human chorionic gonadotropin (HCG), which is commonly used to detect early pregnancy or diagnose gestational trophoblastic disease (GTD) and germ cell tumors,^[4,5]^ is also expressed in malignant tumors, including cancers and sarcomas such as endometrial adenocarcinoma and aggressive osteosarcoma.^[6–8]^ Nevertheless, to our knowledge, no cases of solid pseudopapillary tumors of the pancreas with elevated HCG levels have been reported. Herein, we report a case of solid pseudopapillary tumor of the pancreas with elevated β-HCG levels.

## 2. Case report

A 35-year-old woman was admitted to our hospital with a case of young women for 1 month and vaginal bleeding for >20 days. Her tumor marker (α-fetoprotein, CEA, CA-125, and CA-19-9) levels were within the normal ranges. All other routine laboratory parameters were within the normal range except for positive urine chorionic gonadotropin β and serum β-HCG level of 219.0 mIU/mL. The method we use for detecting β-HCG is enzyme-linked immunosorbent assay.

**Figure 1.**
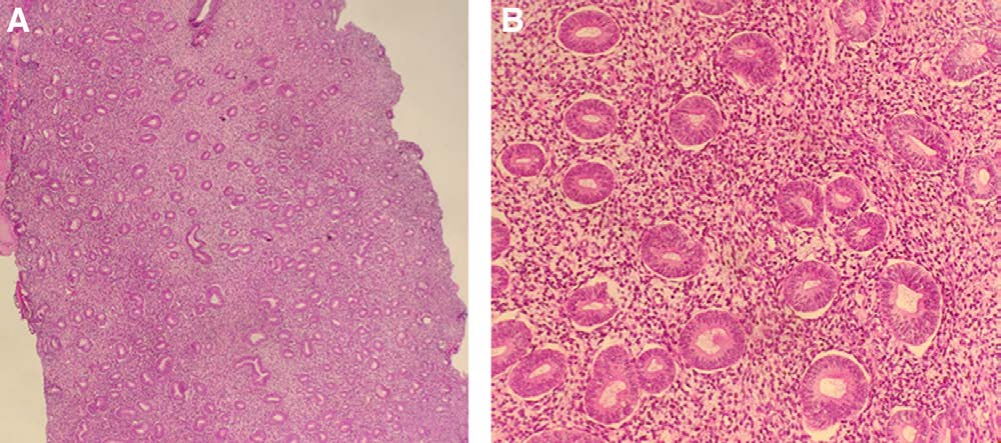
(A, B) Hematoxylin and eosin staining (20×) of the endometrial biopsy sample showing endometrium in the proliferative phase. The stroma also shows a proliferative morphology.

Further examinations were performed to determine the cause of the elevated β-HCG levels. Pelvic ultrasonography revealed no evidence of pregnancy. Subsequently, endometrial biopsy was performed, and light microscopy of the biopsy samples revealed a proliferative phase endometrium, with no decidual changes observed (Fig. 1A, B).

**Figure 2.**
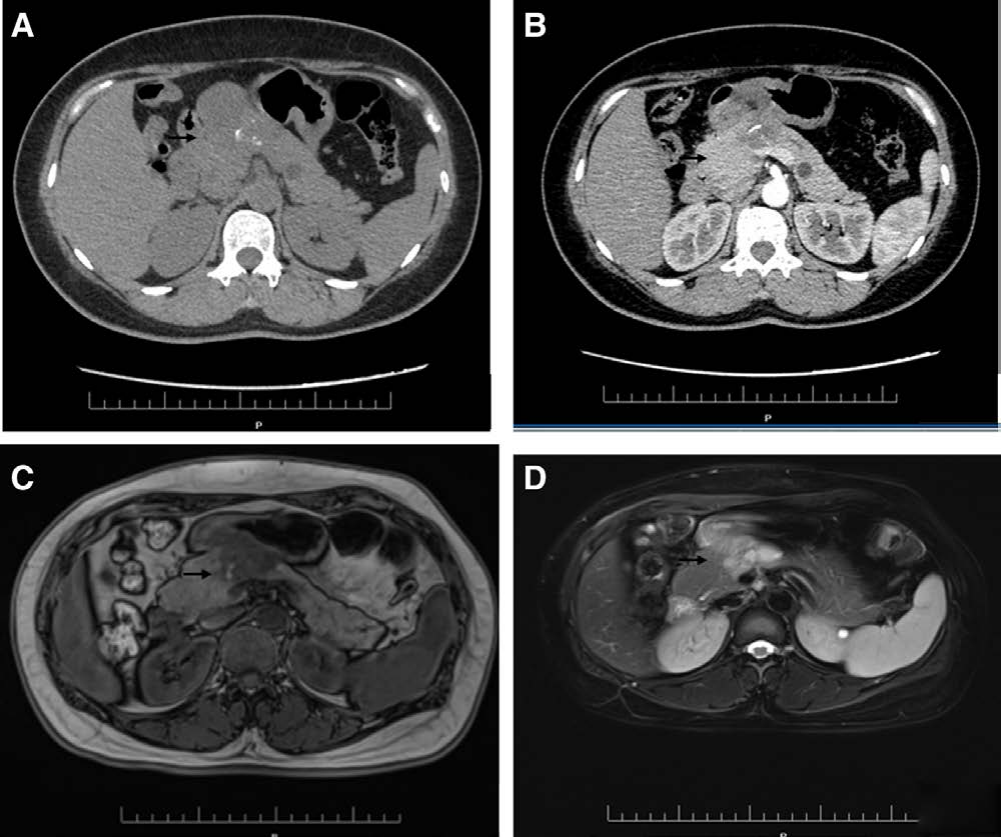
(A) Computed tomography scan of the whole abdomen showing that the shape and size of the pancreas are normal. (B) A mass of soft tissue density shadow is observed in the body of the pancreas; it has clear boundary, the size was 53 mm × 44 mm, the density is uneven, and calcification is observed. The CT value of the solid component was 32 HU. (C) Magnetic resonance imaging shows an abnormal mass of low signal intensity on T1WI and high signal intensity on T2WI in the body of the pancreas, and the lesion size is approximately 54 mm × 52 mm. The signal intensity of the lesion is uneven, and small nodular high signal intensity on T1WI is observed. (D) The lesion shows heterogeneous enhancement on contrast-enhanced scan. CT = computed tomography.

Abdominal ultrasonography revealed a heterogeneous mass in the pancreas that could not be characterized, and computed tomography and magnetic resonance imaging of the abdomen revealed a mass with a multicystic soft tissue density shadow and clear boundary, measuring 53 mm × 44 mm, in the body of the pancreas, suggesting a solid pseudopapillary tumor of the pancreas (Fig. 2A–D).

**Figure 3.**
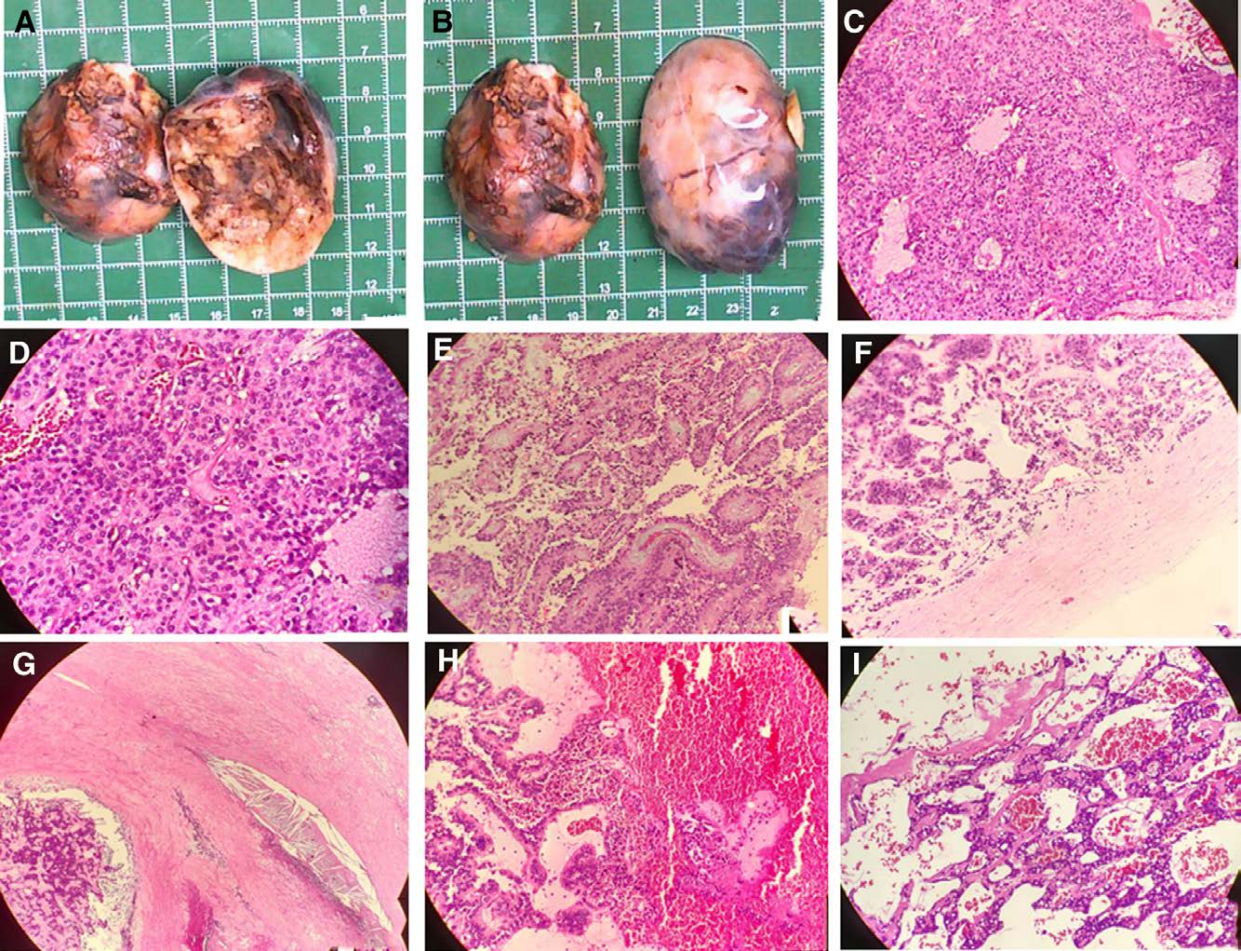
(A, B) A mass, 6 cm × 4.8 cm × 4.2cm in size, is partly encapsulated, grayish-red and grayish-brown cut surface, solid, medium in texture, visible calcification, partly soft in texture, focally cystic. On light microscope, solid (C, D), papillary (E), and pseudoadenoid growth tumors with interstitial fibrosis and hyalinization (F, G) and with hemorrhage and cystic change (H, I) are observed.

**Figure 4.**
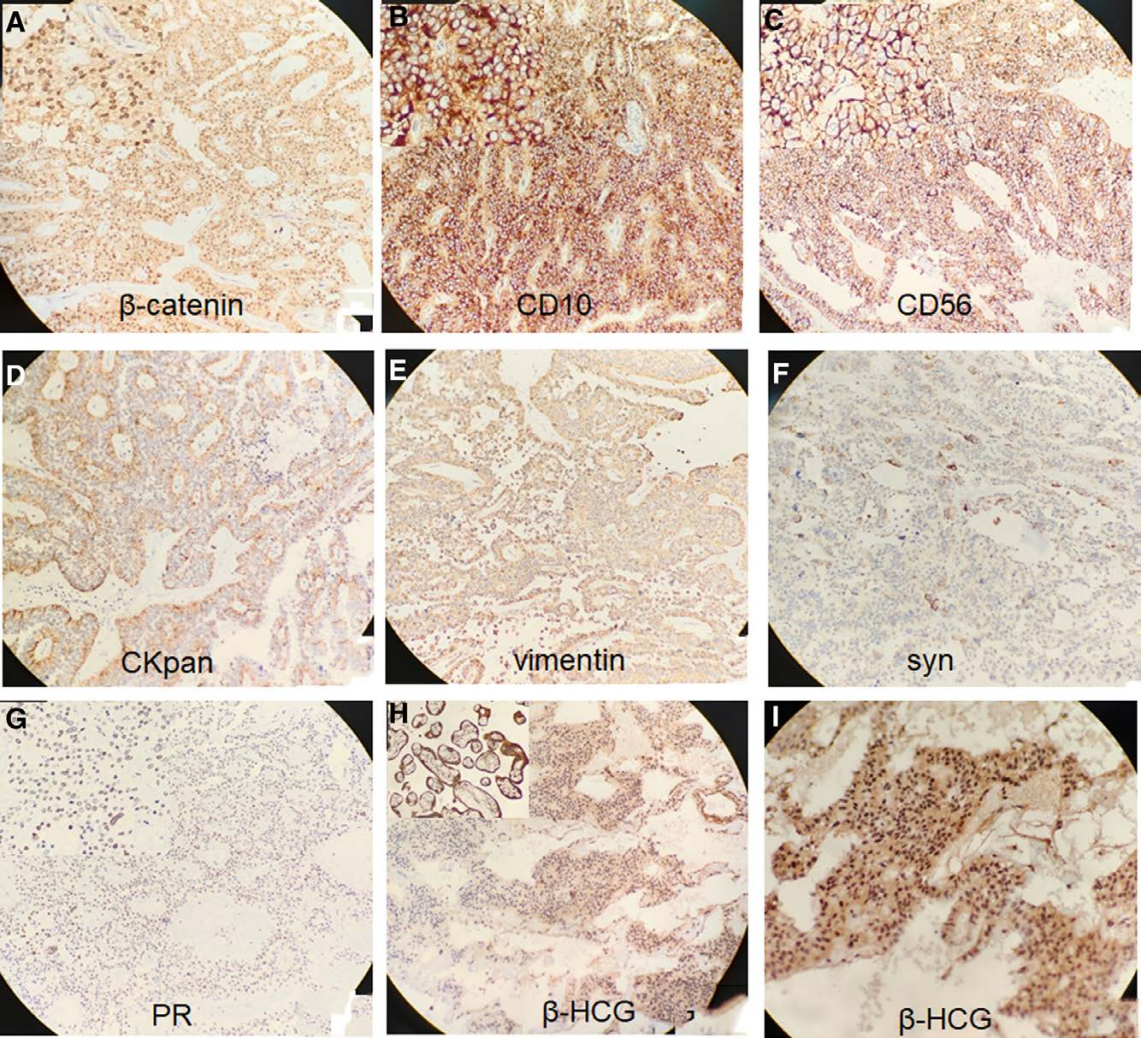
Immunohistochemistry findings: the tumor cells are positive for β-catenin (Nuclear positivity), CD10, CD56, CKpan, and vimentin and focally positive for synaptophysin (syn), (A–I) β-HCG and progesterone receptor (PR). The tumors are negative for AR, chromogranin, CD99, E-cadherin, desmin, and TFE3 (not shown in the figure). AR = androgen receptors, β-HCG = β-human chorionic gonadotropin, PR = progesterone receptor.

Subsequently, surgical treatment was performed. On gross examination, a nodular, friable, partly hemorrhagic and cystic tan mass measuring 6.0 cm × 4.8 cm × 4.2 cm, with partial capsule, was noted (Fig. 3A, B). Light microscopy revealed solid areas composed of uniform small-to-medium, round-to-oval eosinophilic cells with folded nuclei and indistinct nucleoli arranged around delicate fibrovascular septae (Fig. 3C–I). Microscopic tumor cell morphology did not suggest pancreatic ductal adenocarcinoma or pancreatic acinar adenocarcinoma. Immunohistochemistry of the tumor cells was positive for CD56, CKpan, CD10, β-catenin, and vimentin, and focally positive for synaptophysin and progesterone receptors (Fig. 4A–I). The cells were negative for androgen receptors, chromogranin, CD99, E-cadherin, desmin, and TFE3 expression. All immunohistochemical staining in this study was performed using mouse-derived primary antibodies.

Considering these findings, pancreatic neuroendocrine tumor was ruled out, and a pathological diagnosis of SPNs of the pancreas was established.

**Figure 5.**
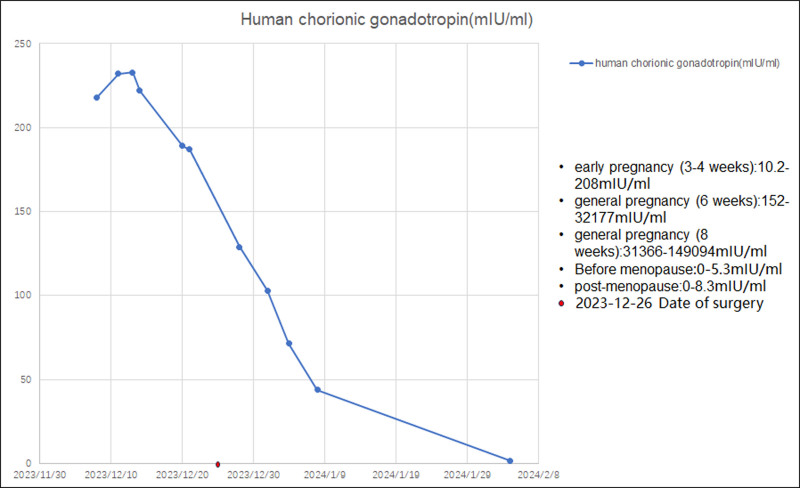
Changes in serum HCG levels from baseline to 1 month after surgery. HCG = human chorionic gonadotropin.

Furthermore, as β-HCG was detected by immunohistochemistry, we ruled out intrauterine and ectopic pregnancies and GTD. Notably, the β-HCG level continuously decreased after surgery and returned to the normal range after 1 month (Fig. 5). Therefore, we speculated that the increased β-HCG levels could be attributed to the exocrine function of the tumor cells. After 6 months of follow-up, the patient recovered well and there was no recurrence.

## 3. Discussion

We present a case of SPNs of the pancreas, which was diagnosed based on hematoxylin and eosin staining and immunohistochemistry results. In addition, β-HCG levels steadily decreased after surgery.

Vaginal bleeding accompanied by elevated serum HCG levels may be associated with various conditions, mainly including: pregnancy-related factors such as normal pregnancy, ectopic pregnancy, and hydatidiform mole, etc. Non-pregnancy factors such as GTD, other malignant tumors (such as germ cell tumors, certain epithelial tumors), etc. This patient is a woman of reproductive age who presented with vaginal bleeding and elevated HCG levels. The first consideration was pregnancy-related diseases. However, after comprehensive examinations (such as ultrasound, dynamic HCG monitoring, pathological examination, etc), the following possibilities were excluded: pregnancy (intrauterine pregnancy, ectopic pregnancy, hydatidiform mole, etc); GTD (such as invasive mole, choriocarcinoma); other endocrine or tumor diseases (such as ovarian tumors, pituitary diseases, etc). The only abnormal finding was a solid pseudopapillary tumor of the pancreas (SPN). After the tumor was removed, the patient’s HCG level returned to normal within 1 month, suggesting that the tumor might be the source of elevated HCG. This case indicates that solid pseudopapillary tumors of the pancreas (SPN) may have the potential to secrete HCG, leading to vaginal bleeding and elevated HCG levels. After excluding common pregnancy and trophoblastic diseases, the possibility of ectopic HCG secretion by non-trophoblastic tumors should be considered, especially tumors in rare locations such as the pancreas.

HCG is a glycoprotein secreted by trophoblast cells of the placenta. It is composed of α- and β-dimer glycoproteins and is normally used to diagnose pregnancy. β-HCG is detected in the urine of pregnant women and patients with trophoblastic disease or cancer.^[9]^ Solid pseudopapillary tumors of the pancreas are exocrine gland tumors of the pancreas, and elevated β-HCG levels due to secretion by such tumors have not been previously reported. Although solid pseudopapillary tumors of the pancreas are locally invasive, they have low malignant potential and often have a good prognosis, even if there is a possibility of metastasis.^[10,11]^ In recent years, cases of spontaneous regression of solid pseudopapillary tumors of the pancreas have been reported.^[12,13]^

A multiple tumor marker protein chip showed that the detection of CA19-9, neuron-specific enolase, CEA, and CA242 is helpful for diagnosing pancreatic cancer. Although CA19-9, CA125, FER, CA242, and CEA levels were increased in benign pancreatic lesions, the expression of HCG did not increase; instead, 25.5% of HCG was increased in pancreatic cancer.^[14]^ We also excluded the possibility of β-HCG secreted by the pituitary gland and other malignant tumors.

Solid pseudopapillary tumors of the pancreas are known to be common in young women, particularly women of childbearing age, and their occurrence in men is rare.^[15]^ Evidence suggests that this prevalence may be related to sex hormones, which also stimulates the study of sex hormone receptors. Yasuda et al.^[9]^ reported a case of spontaneous regression of a postmenopausal SPN of the pancreas, suggesting that changes in female sex hormones may regulate tumor growth. Therefore, we speculated that β-HCG may play a role in the occurrence and development of solid pancreatic pseudopapillary neoplasms.

To date, SPNs appear to be unique to the pancreas; however, their origin is unclear because no clear evidence of ductal, acinar, or endocrine differentiation is available. Some researchers believe that the tumor originates from pluripotent embryonic stem cells with endocrine and/or exocrine differentiation.^[16]^ A single-cell RNA sequencing study of SPNs in children showed that the tumor cells may originate from pancreatic endocrine progenitor cells.^[17]^ Regarding the possibility of endocrine progenitor cells to secrete HCG, pancreatic β-cells have been reported to contain HCG/luteinizing hormone receptors.^[18]^

In conclusion, the cellular origin of SPNs of the pancreas remains an important scientific question warranting further investigation. The elevated HCG levels observed in this case suggest a potential role in tumorigenesis or progression, but the exact mechanism (e.g., whether HCG promotes or inhibits tumor development) remains unclear. Future studies will explore the biological significance of HCG and its receptor in SPNs.

## Author contributions

**Conceptualization:** Minfen Zhang, Zhen Li.

**Data curation:** Lingli Liang, Meiyan Wei.

**Supervision:** Jiao He, Ting Tao.

**Visualization:** Zhi Duan, Hui Chen.

**Validation:** Zhen Li.

**Writing – review & editing:** Wanli Lin.
